# Risk factors associated with *Ctenocephalides felis* flea infestation of peri-urban goats: a neglected parasite in an under-appreciated host

**DOI:** 10.1007/s11250-021-02620-7

**Published:** 2021-02-24

**Authors:** Julia Rose Dahm, Jordana Burdon Bailey, Robert F. Kelly, Patrick Chikungwa, Julius Chulu, Livio Costa Junior, Emily June Freeman, Dagmar Mayer, Stella Mazeri, Neil Donald Sargison

**Affiliations:** 1 University of Edinburgh, Royal (Dick) School of Veterinary Studies, Easter Bush Veterinary Centre, Roslin, Midlothian, EH25 9RG UK; 2 Worldwide Veterinary Service, 4 Castle Street, Cranborne, Dorset, BH21 5PZ UK; 3 Department of Animal Health and Livestock Development, PO Box 2096, Lilongwe, Malawi; 4 Pathology Department, Federal University of Maranhão, São Luís, Maranhão Brazil

**Keywords:** Goats, Malawi, Fleas, Peri-urban smallholder, Conjunctival mucous membrane colour, Zoonosis

## Abstract

Goats are critical in mixed smallholder agricultural systems in lower and middle-income countries, while fleas are important human and animal health concerns around the world. Convenience sampling was used to describe and consider risk factors for flea infestations of peri-urban goats, with the aim of informing the iterative development of animal husbandry and management based control strategies. Seven hundred and ninety-two goats were examined in 228 households across 10 peri-urban communities surrounding Blantyre in southern Malawi. The prevalence of *Ctenocephalides felis* fleas was 18.3, 37.1 and 100% at the levels of individual goats, households and communities, respectively, highlighting a neglected human and animal health concern. Constant introduction of new livestock coupled to a lack of biosecurity within communities, the ubiquitous presence of dog and cat hosts for *C. felis*, the frequency and thoroughness of cleaning overnight goat accommodation, and goat age less than 12 months old were identified as risk factors for flea infestation. This focal cross-sectional study highlights the significance of fleas in peri-urban communities and uncovers trends and commonalities that are needed to inform sustainable disease management. The majority of the peri-urban goat keepers were female, had resided in the same community throughout their whole life and had primary level education. Advice on the planned management of fleas in livestock needs to be tailored towards this demographic group. This approach affords an opportunity to promote public health measures to address household flea infestations and zoonotic disease spread.

## Introduction

Smallholder agriculture involving production of food for both consumption in the home and as a source of income is vital in the world’s poorest countries, including Malawi. Meat production from goats is well suited to smallholder agriculture in many South African Development Countries (SADC) where natural vegetation is often seasonally limited in its quality and availability (Banda et al. 1993; Monau et al. 2020; Sargison 2020). To-date, data pertaining to smallholder livestock systems are inadequate to inform the applied research that is now needed to identify constraints to efficient goat production throughout the region.

Fleas are important parasites of humans, domestic and wild animals around the world, dependent on the correct conditions being present for the completion of each stage of the parasites’ life history. Heavy flea burdens, especially in young animals, can cause debilitating iron deficiency anaemia (Harvey et al. 1982), while a host response to flea bites causes localised inflammation and pruritus. With prolonged exposure to flea saliva, some hosts become hypersensitive, resulting in pustule formation and crusting with intense pruritus (Halliwell 1979); the severity of hypersensitivity induced lesions and pruritis is not proportional to flea burdens. Due to their blood feeding behaviour and ability to move between hosts, flea infestation of animals can also cause potentially severe allergic dermatosis in people working with infested livestock (Soundararajan et al. 2018). Fleas are important as vectors in the biological transmission of viral, bacterial, protozoal and filaroid nematode pathogens (Rust and Dryden 1997; Lappin 2018; Otranto 2018), including zoonotic *Bartonella* spp. and rickettsia. Rickettsial transmission can be transovarial and trans-statial, hence can occur through infestations acquired from newly-emerged pupae. Fleas are also intermediate hosts for the dog tapeworm, *Diphylidium caninum*, acquired by the ingestion of oncospheres by larvae and transmitted when dogs ingest cysticercoid cysts in adult fleas while grooming. Better understanding of the animal health and zoonotic disease risks associated with flea infestation of goats is a global challenge.

Non-penetrating fleas have direct life cycles in which the parasitic adults feed on their hosts’ blood, while the egg, larval and pupae stages are free-living in dark areas on, or under the surface of the ground. Egg hatching is temperature dependent, while the rate and success of larval development depends on the availability of nutrition in the form of concentrated blood, packaged in the faeces of the adult fleas, and of environmental debris. Free-living stages generally require temperatures above 24 °C and cannot withstand major climatic variations. Moulting between the three larval stages and pupa survival also requires adequate humidity. Maintenance of flea infestations, therefore, requires a stable, warm, humid and dirty environment for the free-living stages that is frequently visited by the parasite’s hosts to allow enrichment with the adult fleas’ faecal packages containing host blood, and protected by not being cleaned. Flea transmission occurs when immature adults emerge from pupae and jump onto their hosts or through close contact with infested animals. Most Pulicidae flea species are preferential, but cosmopolitan in their choice of host, hence zoonotic. The prevalence and severity of *Ctenocephalides* spp. flea infestations are, therefore, greatest in those situations where human, companion animal, livestock and/or wild animals hosts regularly spend time in the same stable, protected environment that is conducive for the development and survival of free living stages; for example, where they rest, or are housed at night. Better understanding is needed of how each of these factors relates to goat production in specific settings, including SADCs.

Control of fleas in humans and companion animals usually depends on use of insecticides to kill the adult parasites in association with implementation of hygienic methods to prevent reinfestation from the environment. Organophosphorous pyrethroid compounds can be applied as sprays, dips or shampoos, while fipronil, imidaclopramid or selamectin can be applied conveniently as spot-on treatments, giving up to 3-month protection. Drugs belonging to these groups can be used in small ruminants, but their use is unlikely to be sustainable because no treatments are licensed for goats; *Ctenocephalides felis* could potentially develop resistance (Rust 2016) or tolerance to organophosphate compounds and synthetic pyrethroids; application of these drugs is prohibitively expensive or impractical in smallholder systems; pharmaceutical supply chains are inadequate in most low- and middle-income countries; and drug residues are potentially environmentally damaging. There is, therefore, a need to identify management solutions for the control of fleas in small ruminants, based on understanding of the parasites’ life history and transmission within specific agricultural contexts.

Flea infestations are rare in pastoral livestock kept in cool or temperate climatic zones where environmental conditions may be too cold or unstable for the survival of free-living stages. Most reports of flea infestations in small ruminants emanate from Mediterranean (Yakobson et al. 1981; Yeruham et al. 1989; Christodoulopoulos and Theodoropoulos 2003; Kaal et al. 2006), sub-Saharan African (Obasaju and Otesile 1980; Fagbemi 1982; Opasina 1983), Asian (Soundararajan et al. 2018) and South American (Bezerra et al. 2010) countries, where animals are routinely housed in the same structures each night. Improved understanding of the reasons underpinning differences in the prevalence of fleas in small ruminants between climatic zones, in conjunction with the aforementioned knowledge of flea life histories and their drivers, could help to inform sustainable control strategies.

*Ctenocephalides* flea infestations are known to be common in African small ruminants, albeit there are few reports in the scientific literature accurately describing their species identity, prevalence, or risk factors with reference to the much-needed development of sustainable control strategies. Flea infestation has been reported in 98% of peri-urban dogs in Lilongwe, Malawi (Alvåsen et al. 2016). Fleas have been recognised as a confounding factor in the use of conjunctival mucous membrane colour scoring as an index for the targeted selective treatment of haemonchosis in peri-urban goats in southern Malawi. The aims of this study were to build on these preliminary observations by describing the hitherto unknown prevalence and aforementioned risk factors for flea infestation in peri-urban goats in Malawi, thereby helping to inform more widely applicable sustainable control strategies.

## Materials and methods

### Study site

Livestock production is a relatively small sector within Malawian agriculture, where it is generally extensive and integrated with maize cropping. After chickens, goats are the second most common livestock species kept by 15% of all farming households. The Malawi goat population in 2004 was reported at 1.7 million, with 90% kept by smallholder farmers whose average herd size was reported to be six animals, and maximum 20 (Chintsanya et al. 2004). Goats kept by Malawian smallholders are mostly of an indigenous breed that has evolved and survived in the region with little human intervention. A few improved Boer and Saanen herds were imported to the country during the 1970s and 1980s and as a result, there are now a few crossbred animals, dispersed across the country (Banda et al. 1993).

**Fig. 1.**
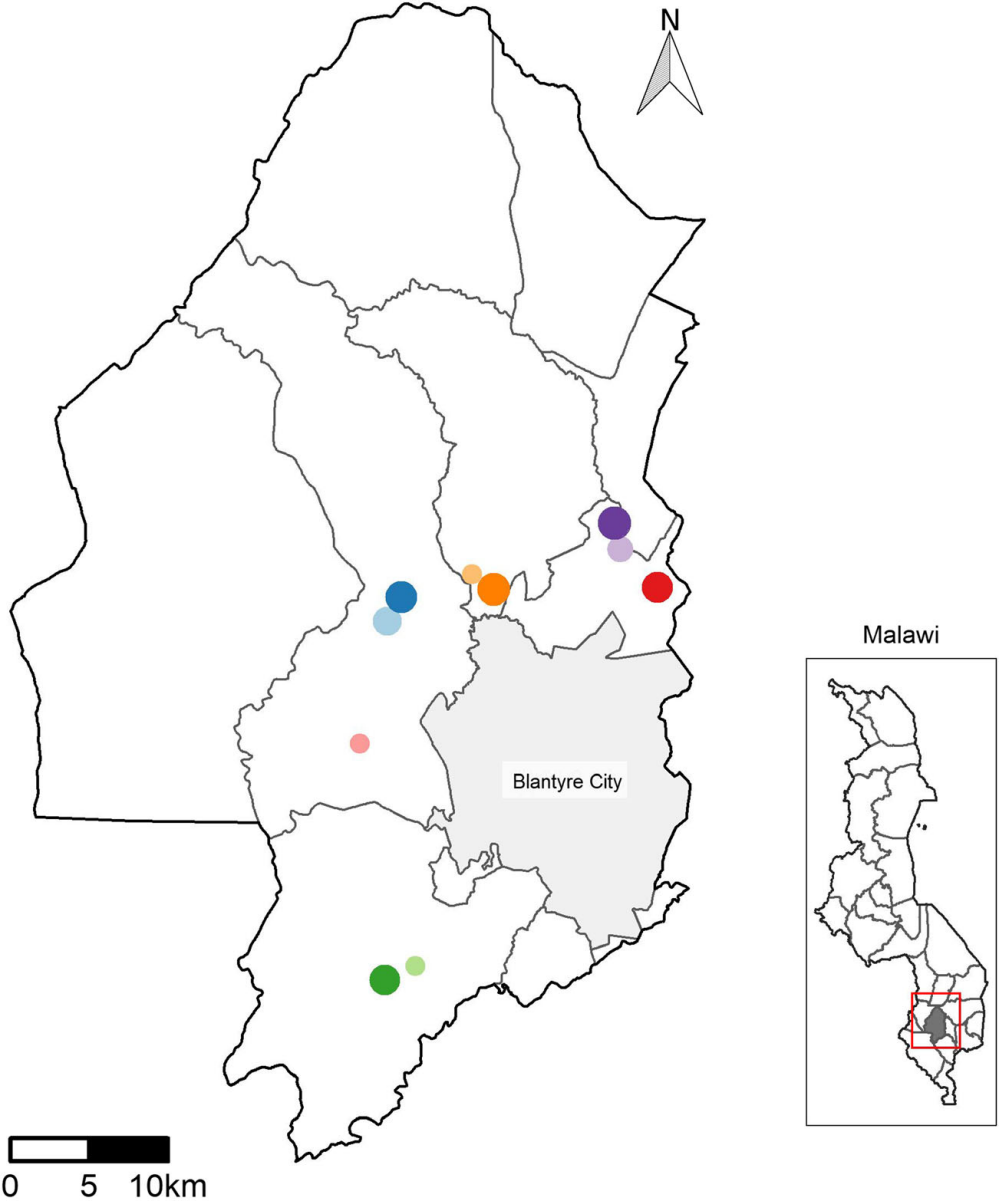
Peri-urban Blantyre sample sites: each site represented by a coloured dot; the size of each dot is proportional to the number of goats sampled

The study was conducted in June and July of 2019, during the middle of the dry season. Ten peri-urban communities were selected within the Blantyre District in the southern region of Malawi. The timing of the study and sites that were visited were chosen to align with a local Mission Rabies (MR) rabies vaccination programme. Within these communities, typically, there are clusters of homes comprising of a small house, another building that serves as the kitchen, and a small outhouse, sometimes with a latrine. Other similar sized structures sometimes serve as goat housing (kholas) and/or chicken coops. Clusters of homes have a shared community space in the middle where the women gather to prepare food, do laundry, socialise and take care of the children. These micro-communities are connected to one another by narrow tracks through cultivated ground, and their boundaries are blurred. Domestic animals generally roam freely, or are tethered across the whole community space. The 10 study sites were geographically separated as shown in Fig. 1.

### Sample size

Malawi has poor infrastructure, lack of adequate communication systems and poor surveillance of highly dispersed livestock populations, with no reliable data on regional goat populations, or community boundaries (Leahy et al. 2017). A pilot survey, conducted in conjunction with a ‘Mission Rabies’ community mass dog rabies vaccination programme during 2018, indicated the presence of 14,585 goats in the Blantyre, Chiradzulu and Zomba districts of southern Malawi. In these districts, the proportion of households that kept goats and the number kept per household was unknown. Although no surveys had been conducted in Malawi previously, fleas had been anecdotally reported as a significant problem in goats. Due to limited availability of population level data and knowledge of likely prevalence of fleas in this setting, non-probability convenience sampling was chosen as the most practical method for this study. Although this method is not the best for applying specific results to a population, it is nonetheless helpful to gain deep understanding of communities and to uncover trends, commonalities and risk factors.

Individual households were sampled in conjunction with the ongoing rabies vaccination programme in the three Blantyre districts. Over the 10-day study period between June and July 2019, the vaccination programme visited one site each day, and the research team travelled door to door to sample as many households as possible per day. Households were geo-tagged, even if no one was present at the time, or they did not possess goats. Sample site boundaries were arbitrary and confined by practicality and timing.

### Goat management practices

Questionnaire data were collected from individual goat keepers, each representing a different household. Questions focussed on demographic information, goat husbandry practices and awareness of ectoparasites and zoonoses. These included specific questions such as ‘what is the primary source of your health advice’; ‘where do your goats stay at night/ during the day’; ‘are their pens cleaned out and if so, how and how frequently’; ‘do the animals scratch or bite their skin a lot’ and ‘do you do anything to control or treat fleas’. Open questions were translated to the local Chichewa language by the translator, and then, responses were recorded in the English language using a bespoke ‘Goat Flea Farmer’ tool in the Worldwide Veterinary Service (WVS) mobile phone-based data recording application (WVS App) (Gibson et al. 2018). The translator was fluent in both English and Chichewa and had worked for MR since 2015. Each interview took approximately 15 min.

### Clinical examination

Goats from each smallholder were restrained to allow immediate evaluation of the colour of the conjunctival mucous membranes using a FAMACHA© card (Malan et al. 2001). The conjunctival colour was classified from red (score 1), through red-pink (score 2), pink (score 3) and pink-white (score 4) to white (score 5). Where possible the FAMACHA scoring was conducted in direct sunlight. Each animal was body condition scored (BCS) by palpation of the lumbar vertebrae with a range of 1 (thin) to 5 (fat) as described by Villaquiran et al. (2004), extrapolating from a system developed for European sheep breeds (Russel 1984). The age and sex of each animal provided by its keeper were confirmed by examining its incisor tooth dentition (Eubanks 2012) and external genitalia. Animals were observed for clinical signs associated with fleas. Scores 0–2 were used based on a system of scoring developed for flea infestations of dairy goats in Greece (Christodoulopoulos et al. 2006) and amended to account for differences in flea species, goat breed, region and time spent observing the goats. When observed, a score of 0 was given if no restlessness, rubbing or chewing was observed; a score of 1 was given if some restlessness, rubbing and chewing was observed; and a score of 2 was given if cut hairs, alopecia and/or redness was observed. Finally, a human ‘nit comb’ was used to comb twice through the hair covering each of the inguinal and axillary areas on both sides of each goat. The numbers of fleas seen on the comb were noted for each of the four areas. This specific combing protocol, ‘Flea Burden Grading’, was first developed in a pilot study in order to be repeatable and reliable throughout the data collection process. All of the clinical data were recorded using a bespoke ‘Goat Flea Goat’ tool in the WVS App (Gibson et al. 2018).

### Data collection process

The data collection process was first piloted on 20 smallholder goat keepers to ensure optimal practicality and acceptability; these responses are not included in the results. Changes were made according to what was actually feasible in the field (i.e. weighing the animals was removed from the protocol due to inability to accurately gather correct weights in an efficient and stress-free manor; gathering clinical data from animals other than goats was also abandoned due to time constraints and the priority to adhere to the specific aims of this study). For the main study, data were collected over a 10-day period between June and July 2019. Each sampling day involved a separate sample site designated by geographic location. The data gathering process was as follows: the translator and researcher would begin in a new geographic area (sample site) at the start of a sample day. The ‘path tracker’ function from the WVS App (Gibson et al. 2018) was used to record the global positioning satellite (GPS) locations of all the homes that were visited and of the continual path that the research team took. Each home was approached, and the residents were asked by the translator if they had goats and were willing to participate in the study. The research team asked to interview the primary goat keeper from each home. Children under the age of 18 were not included per University of Edinburgh Human Ethics Review Committee stipulation, and if the primary goat keeper consented, the translator would begin with the ‘Goat Flea Farmer’ questionnaire function, asking and recording the answers to all of the questions in the WVS App (Gibson et al. 2018) under the specific anonymous participant identification code (ID). The researcher would then fill out the ‘Goat Flea Goat’ clinical data survey as completely as possible for each goat belonging to the smallholder that could be caught, safely restrained and examined, using specific goat IDs that corresponded to the specific participant IDs in the ‘Goat Flea Farmer’ questionnaire. The ID system gave each village a letter, starting with B (A was used for the pilot day). Each household was given a number starting with 1, and each goat was given a number starting with 1. Community or village names were recorded when known. The translator and researcher both used the same ID system so that smallholders could be linked with their goats. At the end of each day, the data were uploaded from the mobile phones to the WVS App’s main server, from which they could be accessed and downloaded into Excel spreadsheets (Microsoft Corporation, USA).

### Statistical analyses

All statistical analyses were performed using packages and functions in R Studio version 1.1.463 (https://cran.r-project.org/) (R Core Team 2020). Graphics were produced using the ‘ggplot2’, ‘tidyverse’, ‘dplyr’, ‘gridExtra’ and ‘lattice’ packages. Spatial data were displayed using ‘rgdal’, ‘ggthemes’, ‘sf’, ‘ggrepel’, ggmosaic’ and ‘RColorBrewer’ packages, and shape files were obtained from the GADM database of Global Administrative Areas (www.gadm.org).

Descriptive household and goat survey results were initially reported as counts, proportions and bar plots. For the goat survey, relationships between presence of fleas and potential risk factors were initially explored using bar plots. Interesting relationships were subsequently tested using *t*-test (for numeric data) or chi-squared test (for categorical data) assuming independence. Respectively the ‘t.test’ and ‘chisq.test’ functions were used in the stats package (R Core Team 2020). Unless otherwise stated, results with a *p* value ≤ 0.05 were considered to be significant.

## Results

### Explanation of descriptive analyses

In total, 792 goats were examined, and questionnaires were completed from 228 smallholders. On the rare occasions when some of the goats belonging to the smallholder could not be caught or safely restrained, these animals were omitted from the ‘Goat Flea Goat’ clinical data entry. Likewise, there were occasions when a whole group of goats belonging to a specific home were away at the time that the researcher was present, mostly taken to another location to graze. In these cases, the ‘Goat Flea Farmer’ questionnaire was completed, but corresponding ‘Goat Flea Goat’ clinical data were not recorded. There were also a few goats for which the corresponding “Goat Flea Farmer’ questionnaire could not be completed, as the primary keeper was not able to be interviewed. In total, data were collected from 213 smallholders for 785 goats. Analyses describing lower numbers reflect missing data for the aforementioned reasons.

### Household-level husbandry practices

Overall, the number of goats per household ranged from 0 to 19, with a mean of 4.0 and median of 3.0. Fifty-one of the goat-owning smallholders (22%) also had dogs; 128 (56%) kept chickens; six (2.6%) kept cattle; 60 (26%) had cats; one kept sheep; 24 (10%) kept pigs; three (1.3%) kept ducks and three (1.3%) kept pigeons. A 71.4% of the 213 people who participated in the interview were female. Out of the 211 primary goat keepers who reported their age range, 11.4% were under 25 years old, 74.4% were between 25 and 60 years old and 14.2% were over 60 years old. A 62.9% of the smallholders stated they only had a primary school education, 24.4% had no education and 12.7% had completed secondary school (with one person having further education). Two hundred and ten participants (98.6%) had resided in their current community for their whole life.

When asked about sources of animal health advice, 1.4% cited their community or family; 3.3% referred to a vet (meaning a paraveterinary Assistant Veterinary Officer (AVO), or vet scout (now referred to as Animal Health Surveillance Assistants), as described by Leahy et al. (2017); and 95.3% said that they did not receive any animal health advice, or were unsure.

Most goats were kept outside during the day (98.1%) with the majority of smallholders keeping them on a rope tether (loop) tied to a stake or tree (87.6%) throughout the whole year. Only three of 209 smallholders (1.4%) kept their goats in an outdoor khola during the daytime (*n* = 209). Most (78.8%) of the smallholders kept their goats in their family home overnight, with the rest in raised kholas (20.3%) or in the separate kitchen building (0.9%).

A 74.6% of households acquired replacement goats from sources other than their own breeding. These sources included gifts from friends, family or other people in the community (22.5%); animals bought at a market (50.2%); and animals provided by an organised NGO or Government scheme (1.9%). A 52.1% of smallholders reported selling their goats to support family income. Most of these 111 smallholders who sold their goats did so at markets (trading posts, where goats are traded alongside other produce and goods, often adjacent to a slaughter slab) (96.4%); 1.8% sold privately to neighbours; and 1.8% sold their animals by both methods.

**Fig. 2.**
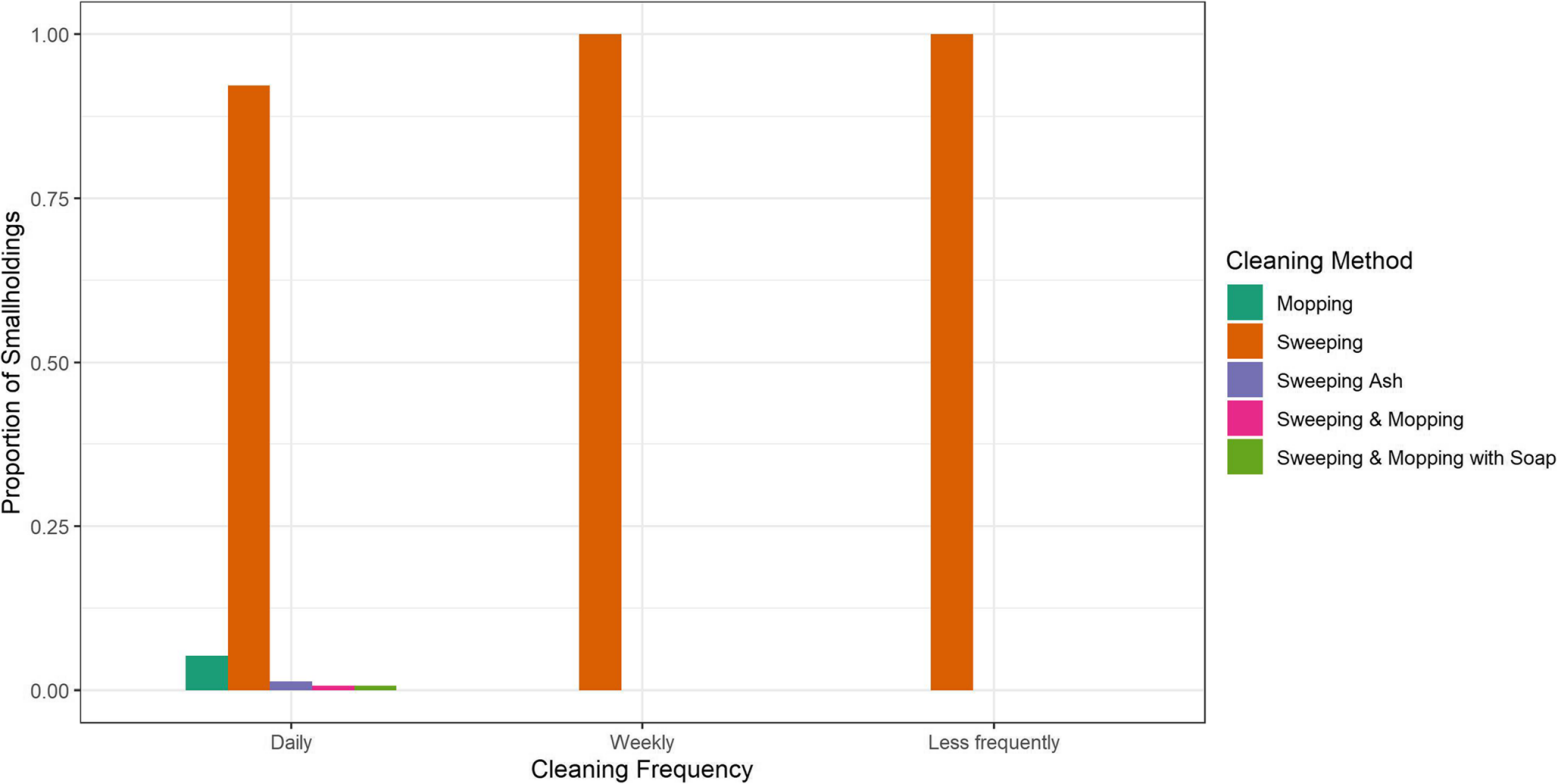
Cleaning frequency and methods used by 210 smallholders for their goat accommodation as a possible risk factor for flea infestation. (From the 213 respondents: one did not know and two reported not cleaning). Two participants reported not cleaning at all. From those who did clean, the two main methods used were sweeping (*n* = 200) and mopping (*n* = 8), albeit the nature and rigour of how these tasks were undertaken could not be determined. Those who reported cleaning methods other than, or in addition to sweeping also cleaned more frequently

Smallholders’ responses about the nature and frequency of cleaning the goat accommodation are shown in Fig. 2.

The two most frequently reported goat husbandry challenges were diseases (27.2%) and lack of feed (33.8%), although 20.2% did not report any problems. Death of goats was also considered to be a significant problem (15.5%).

Questions were asked about knowledge and awareness of fleas (which the Chichewa language did not necessarily differentiate from other visible ectoparasites, such as lice and ticks) on the smallholder’s animals (due to confusion arising from translation, this may not have discriminated between goats and other species), including treatment and control options. A 39.0% of the smallholders believed that the main impact of ectoparasites on their animals was weight loss, and 28.6% thought it was hair loss. 10.3% attributed ‘bad health’; 10.3% attributed ‘disease’; 10.3% attributed ‘no impact’; 1% attributed ‘lack of feed’ to ectoparasites and 0.5% responded ‘unknown’. A 74.2% of the 213 smallholders interviewed did not perceive any problems with ectoparasites on their own animals. Eighteen smallholders in whose goats’ fleas were identified perceived associated problems, while 61 smallholders in whose goats’ fleas were identified did not perceive any associated problems.

The smallholders were asked the questions: ‘do you do anything to control fleas’ and ‘do you do anything to treat fleas’, independently. However, the responses for both were interchangeable (reflecting difficulty in translating the different meanings of treatment and control), therefore, an answer other than ‘nothing’ for either question is reported as having some form of control. While the majority (94.4%) did nothing, 5.6% of 213 respondents reported having used either ‘topical products’, ‘dips’ or ‘pesticides’ (although information concerning the timing and animal species involved was lost in translation). None reported animal husbandry methods for the management control of fleas, albeit this could have been due to misunderstanding in translation of the question.

An 80.3% of the 213 smallholders reported not having experienced any human health risk associated with flea infestation of their animals, whereas 16.4% reported the family experiencing flea bites and 2.8% reported itching. One smallholder reported both bites and itching.

### Goats and clinical signs

The results of the ‘Goat Flea Goat’ clinical data survey are outlined in Table 1. Analysis of the presence of clinical signs (scores 1 and 2) and FAMACHA© scores of 3, 4 or 5 (representing slight to severe anaemia) by age group is shown in Table 2. There was a trend towards higher FAMACHA© scores in animals in poorer BCS (Table 3).

**Table 1.** Clinical data gathered from 785 goats on 213 smallholdings

		Number of goats	Percentage
Sex		*n* = 781	
	Entire male	173	22.2
	Female	608	77.8
BCS (1–5)		*n* = 783	
	1.0	12	1.5
	1.5	50	6.4
	2.0	127	16.2
	2.5	212	27.1
	3.0	360	46.0
	3.5	22	2.8
Age		*n* = 780	
	2 weeks to 12 months	349	44.7
	12–18 months	108	13.8
	18–30 months	55	7.1
	30–42 months	83	10.6
	Over 3.5 years	185	23.7
FAMACHA© score		*n* = 783	
	1	49	6.2
	2	212	27.1
	3	259	33.1
	4	203	25.9
	5	60	7.7

There were no goats with BCS greater than 3.5

**Table 2.** Numbers [%] of goats with clinical sign scores 1 and 2 and FAMACHA© scores 3, 4 and 5, broken down by age of goat

	Clinical signs		FAMACHA©		
	1	2	3	4	5
2 weeks to 12 months	29 [52.7]	3 [50.0]	100 [38.6]	76 [37.4]	38 [63.3]
12–18 months	8 [14.5]	2 [33.3]	39 [15.1]	23 [11.3]	5 [8.3]
18–30 months	1 [1.8]	0	19 [7.3]	15 [7.4]	2 [3.3]
30–42 months	5 [9.1]	0	32 [12.4]	21 [10.3]	4 [6.7]
Over 3.5 years	12 [21.8]	1 [16.7]	69 [26.6]	68 [33.5]	11 [18.3]
Total	55	6	259	203	60

A 92.0% of the 785 goats that were examined received a clinical signs score of 0

**Table 3.** Body condition score in relation to FAMACHA© score by individual goat (*n* = 781)

FAMACHA© score	1	2	3	4	5	Total
BCS						
1.0	0	0	0	3	9	12
1.5	0	5	10	17	18	50
2.0	1	26	35	52	13	127
2.5	13	64	70	54	11	212
3.0	34	103	138	74	9	358
3.5	1	12	6	3	0	22
Total	49	210	259	203	60	

### Flea prevalence and risk factors

**Fig. 3.**
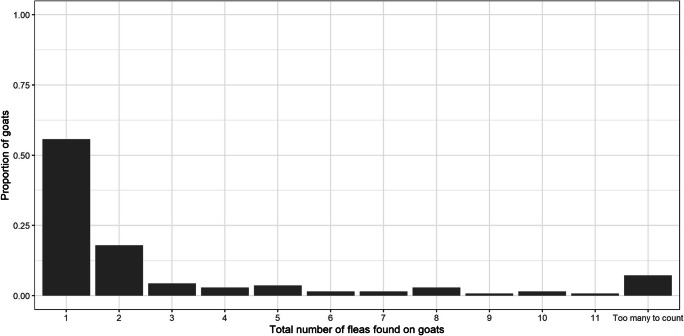
Distribution of relative flea burdens, based on the total numbers of fleas identified using the combing method

Fleas were found on goats kept in each of the 10 peri-urban communities. Reference to published keys (Lawrence et al. 2019; Linardi and Santos 2012) confirmed their identity as *C. felis*. Fleas were found on goats in 79 (37.1%) of the 213 households visited. One hundred and forty (17.8%) of the 785 goats examined using the ‘Flea Burden Grading’ method were positive for fleas. The distribution of flea counts is shown in Fig. 3.

**Fig. 4.**
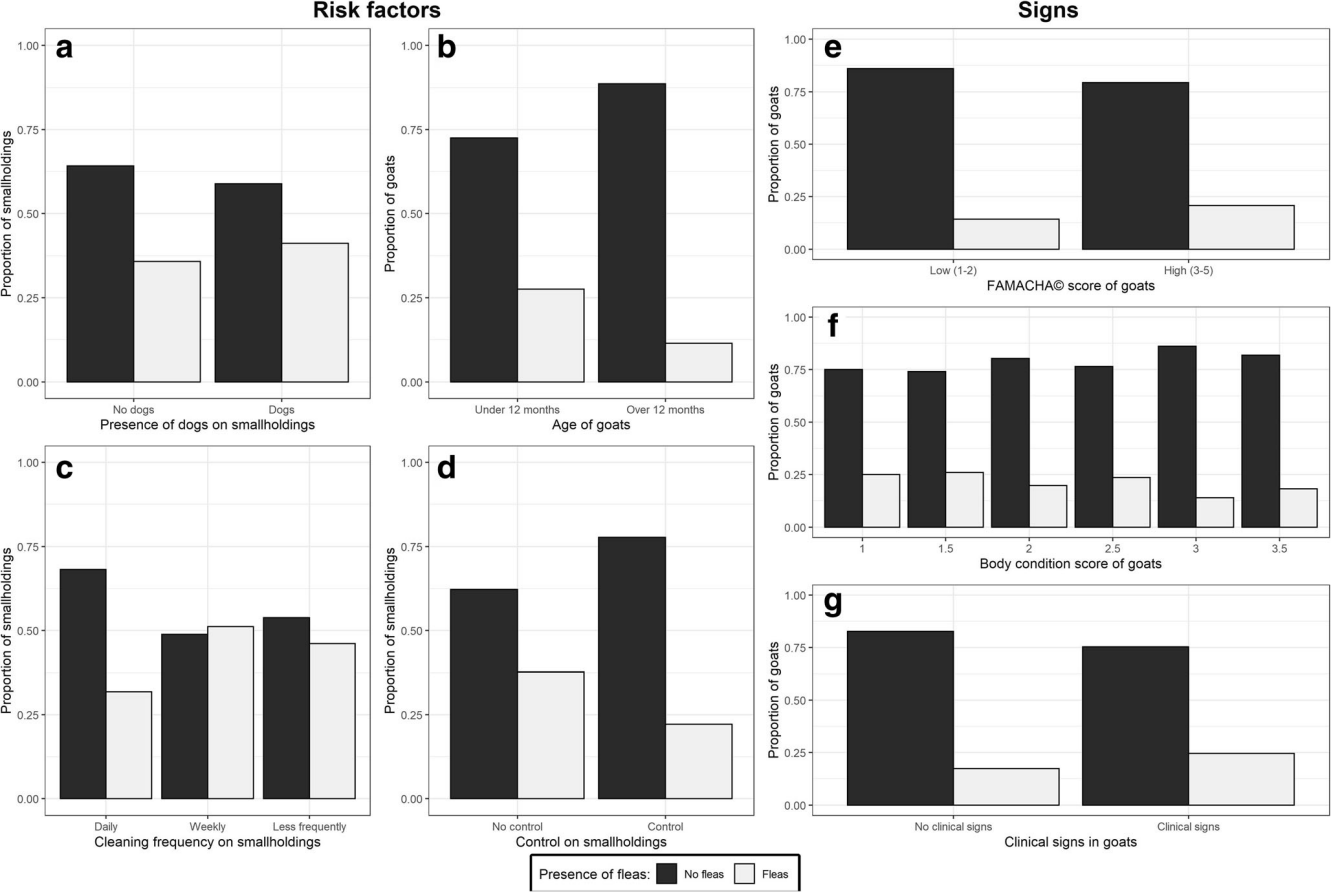
Different risk factors for flea infestation in goats. Flea presence for all is designated by the colour of the bar. Variables are shown on the *x*-axis and proportions of goats or households out of 100% are on the *y*-axis. (**a**) Proportions of smallholders who reported having dogs in relation to the presence of fleas found on any goats in the smallholding (*n* = 213). Having dogs was not significant as a risk factor for fleas in goats. (**b**) Age of goats grouped by under 12 months and over 12 months with respect to presence of fleas per individual goat (*n* = 780). The prevalence of fleas on goats less than 12 months old (26.9%) was significantly higher than on goats older than 12 months old (11.0%) (*p* value < 0.001). (**c**) Cleaning frequency in relation to presence of fleas on smallholdings. [Cleaning frequency was originally reported as twice a day, every day, every other day, twice a week, once a week, twice a month, once a month, every 3 months, every 6 months and once a year (*n* = 210). These were collated into daily (twice a day, every day, every other day and twice a week), weekly (once a week, twice a month) and less frequently (once a month, every 3 months, every 6 months and once a year)]. The percentage of goats with fleas (31.8%) was lower where daily cleaning was practiced than where weekly (51.2%), or less frequent (46.2%) cleaning was practiced. (**d**) Control and treatment methods were looked at with presence and absence of fleas on the smallholding (*n* = 213). There was a reduction in percentage of goats with fleas where smallholders reported using some type of control/treatment method (22.2%) compared to nothing to control/treat fleas (37.7%), but the group size of the former was too small to attribute statistical significance. (**e**) Proportion of individual goats who had fleas in respect to FAMCHA© score (*n* = 780). The difference was not significant. (**f**) Proportion of individual goats who had fleas in respect to BCS (*n* = 783). The difference was not significant. (**g**) Proportion of individual goats who had fleas in respect to clinical signs (*n* = 762). No significance could be inferred because the overall percentage of goats that were positive for clinical signs was too small, confounded by the potentially low sensitivity of the diagnostic method

Risk factors for the presence or absence of fleas, and at individual goat or household levels are shown in Fig. 4.

## Discussion

Fleas were found on 17.8% of 785 individual goats belonging to southern Malawian peri-urban smallholders that were examined during this study. The prevalence of flea detection was higher (37.1%) when taken at the level of the 213 households, representing groups of goats kept overnight in close confinement to each other; and at the level of communities (100%), representing animals that would come into contact with each other and with other potentially flea infested hosts and environments during the daytime. It is inconceivable that the fleas would not spread on to and between each of the animals within groups housed together at night; hence, the higher prevalence estimate at household level implies that the sensitivity of the detection method involving combing twice in four sites over the body was low. Previous studies involving dogs recommended combing for 5 min over the whole body in order to detect high proportions of adult fleas that were present (Zakson et al. 1995; Durden et al. 2005). However, the time and restraint essential for this approach would have been impractical in the context of the aims of this study. The results are nevertheless informative, indicating that the actual prevalence of fleas in the goats could have been between 37.1 and 100%, representing a serious and neglected animal and human health concern for southern Malawi’s peri-urban livestock keepers. The relationship between number of fleas identified and the burden size is unknown; nevertheless, the data may give an indication of the relative distribution of flea counts between animals.

Risk factors for flea infestation were selected on the basis of the research team’s understanding of the drivers of the parasites’ life history (Rust 2017). Statistical significance was not attributed to all of the risk factors that were examined, due to small numbers of households or goats undertaking particular practices, and the low sensitivity of the method of flea detection. Conducting a stratified cross-sectional survey across the districts would have been beyond the limitations of the convenience sampling method used in this study due to limited access to population level data in this setting. As flea burdens are likely to be environmentally dependent, future surveys should be conducted longitudinally to assess flea epidemiology throughout the wet and dry seasons. Improving the sensitivity of diagnostic techniques would also be useful to estimate presence and distribution of fleas within a host or household. Nevertheless, these results reveal trends and allow the identification of probable risk factors in accordance with the principles of iterative planned animal health management; the next step in the theory of change being to monitor the impact of management to address these factors (Sargison 2020).

Most of the goats were kept in the same environments as dogs and cats, for which *C. felis* has a high affinity (Lawrence et al. 2019). Most were also kept in the same environments as other livestock which have also been observed to be infested with various flea species, including *C. felis* (Braae et al. 2013). However, ownership of alternative flea host animals was not shown to be a risk factor for flea infestation in goats in this study; possibly because the nature of the peri-urban communities in which the goats were kept would have meant that few if any did not come into regular contact with environments frequented by free-roaming dogs, cats, chickens and sometimes pigs.

Most households acquired goats from sources other than their own breeding, contributing to the generally poor biosecurity, and being a risk for the introduction of fleas. Most of the goats were tethered outside on waste ground, along roadsides or on the margins of cultivated land during the day, and housed at night in, or close to the family home (Banda et al. 1993), primarily to protect against theft. Of the goats kept within the family home, some were kept in specific rooms, sometimes accessed through a hole in the wall, while others were kept in the same room as occupied by the household, with a clear risk of transmission of zoonotic disease. Intuitively, flea infestation was lowest where daily cleaning was practiced, which would have removed free living flea stages, and their nutritional enrichment with the adult fleas’ faecal packages containing concentrated host blood.

The majority of the peri-urban smallholders in this study showed a lack of awareness of the presence, importance and impacts of flea infestation of their livestock, of public health consequences. None was aware of the principles of management control of fleas based on understanding of the drivers of their life history, while only 5.6% reported having ever used drugs for flea control. This highlights an educational need. The majority of the primary goat keepers were female, as previously reported (Monau et al. 2020) and between 25 and 60 years old, and most had resided in the same community all of their lives and only had primary school education. Hence, a priority for flea control in goats is to identify advice on practical management strategies to reduce the risk of introduction and establishment of heavy flea infestations. This must be appropriately targeted towards the needs and availability of this demographic group, involving community leaders to take into account the main source of advice being other smallholder livestock keepers (Hopker et al. 2018).

Based on the results of this study, education pertaining to the practical management of flea infestation in African goats should address: preserving the use of insecticidal drugs for the strategic targeted treatment of fleas in primary animal hosts and humans; the importance of regular and thorough cleaning of overnight accommodation; biosecurity measures to reduce the level of introduction of new infestations to a community; and emphasis on the potential importance of fleas as vectors of animal and human diseases. Consideration of control of flea infestations in livestock affords a much needed opportunity to raise awareness of the importance of fleas in humans; and of the need for control at both household and community levels.
